# Evaluation of the Accuracy of a System to Align Occlusal Dynamic Data on 3D Digital Casts

**DOI:** 10.1155/2018/8079089

**Published:** 2018-06-06

**Authors:** Iñigo de Prado, Mikel Iturrate, Rikardo Minguez, Eneko Solaberrieta

**Affiliations:** Department of Graphic Design and Engineering Projects, University of the Basque Country (UPV/EHU), Spain

## Abstract

In recent years the T-Scan system has introduced the possibility of importing digitization of dental arches to its registrations. This is a remarkable advance, which allows an intuitive display of the location of the gathered dynamic data on the denture. Nevertheless, today's usual method of manually positioning the arch in relation to the T-Scan's force registration gives rise to the possibility of human error. In order to guarantee a good alignment between the dynamic registration and 3D digital casts, a specific method was developed. The aim of this study is to evaluate the accuracy of this alignment method. For this purpose, it was compared with the most common procedure for detecting occlusal contacts, the articulating paper method. The comparison comprised overlapping digital models of both methods. Contacts of casts of 11 adults were registered, both with articulating paper and the T-Scan system. For one method, articulating paper marks were scanned in color; for the second method, the previously mentioned alignment was carried out with the T-Scan registrations. The results of both methods were overlapped in 3D digital casts, quantifying occlusal data matches. Statistical analyses were made to measure the quality of this alignment method. The study revealed a mean matching percentage of 79.02%, confirming the high reliability of the method.

## 1. Introduction

Although several technologies have been developed for finding occlusal contact areas and the occlusal pressure on each, such as waxes, pressure sensitive films, and occlusal sprays [1], the use of articulating paper is the most common practice, even in today's digital era, as it is easy to use and effective [2]. However, although its validity is widely approved by the dental community, some studies reveal that the marked area can be affected by some variables such as saliva and slippery surfaces [1, 3–6].

In addition, it seems that the deduction of the applied force based on the marked area size is questionable. Apparently, there can be a correlation between the force on teeth and the marked area size, but there are several reasons for believing that the clinician's interpretation is also highly significant and can lead to an incorrect diagnosis [7–10].

In this context to achieve reliable data of those occlusal forces, an electronic device called T-Scan has been in use for some decades now. This device is capable of detecting pressure thanks to a flexible sensor with an electronic circuit made with conductive ink. This sensor sends the gathered information to a computer, which shows the contacts in a way that can be intuitively interpreted: they are represented on a colored map with a certain range of colors, with each one representing a different force intensity. All the contacts (colored according to the pressure each point receives) are accompanied by a percentage number, which shows the force distribution along the dental arch. Moreover, it shows not only the contacts of a particular moment, but also a timed record in which the user can see the development of the bite: the first contact, the maximum intercuspation, the applied maximum force moment, the last contact, etc., whereas with articulating paper it is impossible to know if a mark has been made by a momentary contact or not [11]. This idea of force detection makes it possible to consider the occlusal dynamics of patients in both diagnosis and subsequent treatment [12].

In recent years the Tekscan Company has released new versions of T-Scan's software, which allow the importing of digitized scans of dental arches into the program. This gives a much richer interpretation of the dynamic data because it is easier to appreciate finer details such as knowing with more accuracy which tooth receives a specific contact. In addition, the visualization is much more attractive and it enables improved understanding.

However, the positioning of the dental arch in relation to the T-Scan registration must be made manually. This makes it possible for alignment deviations to occur.

That is why a digital alignment method has recently been developed [13]. This procedure uses reference points to create a correlation between physical casts and T-Scan's dynamic data, enabling a nonmanual and more exact alignment.

Although several studies which test both articulating paper and T-Scan have been undertaken, even some comparing both [14–18], their results with regard to the contacts' location accuracy have never been compared digitally. In fact, to complete such comparison, it was necessary to obtain digital 3D objects (due to the impossibility of comparing digital and physical objects).

This study measured the accuracy of this alignment, comparing the resultant contacts' position with those obtained with articulating paper, as it is broadly accepted as an exact method for locating contacts.

## 2. Material and Methods

Complete casts of 11 healthy adults were used for this study. All were placed on articulators and manipulated by only one technician.

**Figure 1:**
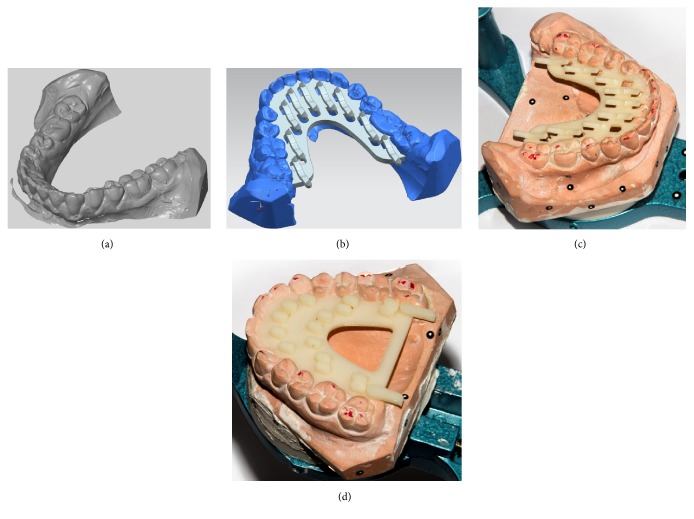
Manufacture of oral reference appliances. (a) Digitization made with ATOS Compact Scan. (b) Design of oral reference appliance. (c) 3D printed device placed on mandible. (d) 3D printed device placed on maxillary cast.

Firstly, the method developed by Solaberrieta was carried out [13]: the 11 casts were digitized using an ATOS Compact Scan (GOM) (Figure 1(a)). Then, drawing on these scans, auxiliary appliances were developed; these devices were designed for marking some reference points on the T-Scan during the bite, in order to have a direct relationship between the information detected by the software and the actual casts (Figure 1(b)). They were printed with a Dimension Elite 3D printer, in ABS plastic. Then they were accurately placed in casts (Figures 1(c) and 1(d)). In this phase of the experiment, it was necessary to use a highly accurate scanner because these oral appliances need to fit perfectly in their corresponding casts.

**Figure 2:**
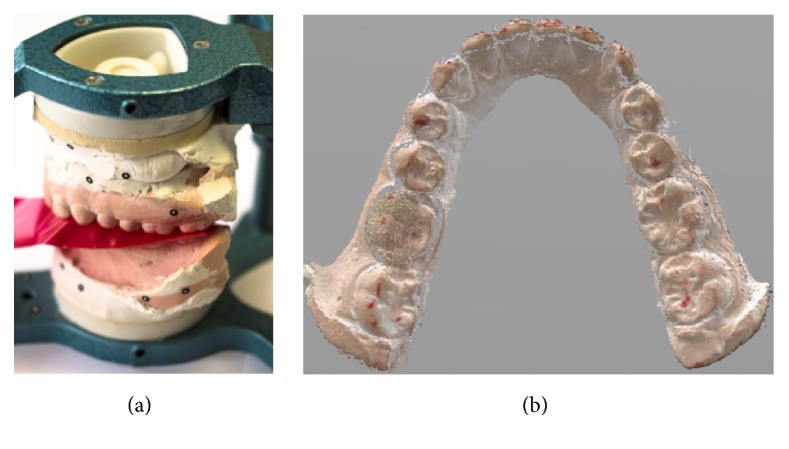
Gathering of occlusal contacts through articulating paper method. (a) Contacts marking with an Arti-Fol 8 *μ*m articulating paper. (b) Scanned cast by a Go!SCAN 3D.

Secondly, an Arti-Fol 8 *μ*m thick articulating paper was used for locating contacts on all casts (Figure 2(a)). Once the teeth were marked, a Go!SCAN 3D (Creaform) scanner was used to acquire colored digital casts (Figure 2(b)). These digitizations were aligned with those acquired in the previous step by best fit functions, using a mesh-treatment specific program (Geomagic Studio 2014).

**Figure 3:**
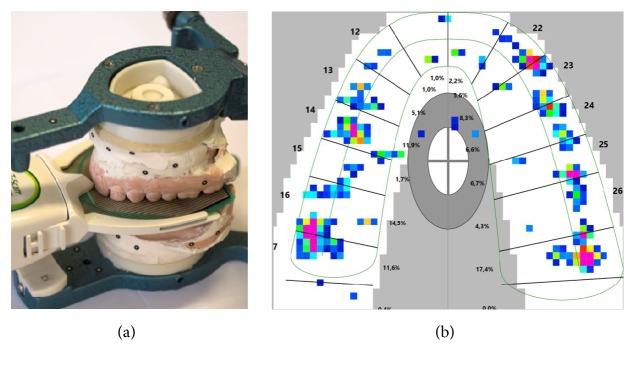
Gathering dynamic data with the T-Scan. (a) Use of T-Scan, having previously placed the reference appliances. (b) Image of the maximum intercuspation of T-Scan, where reference contact points are visible.

**Figure 4:**
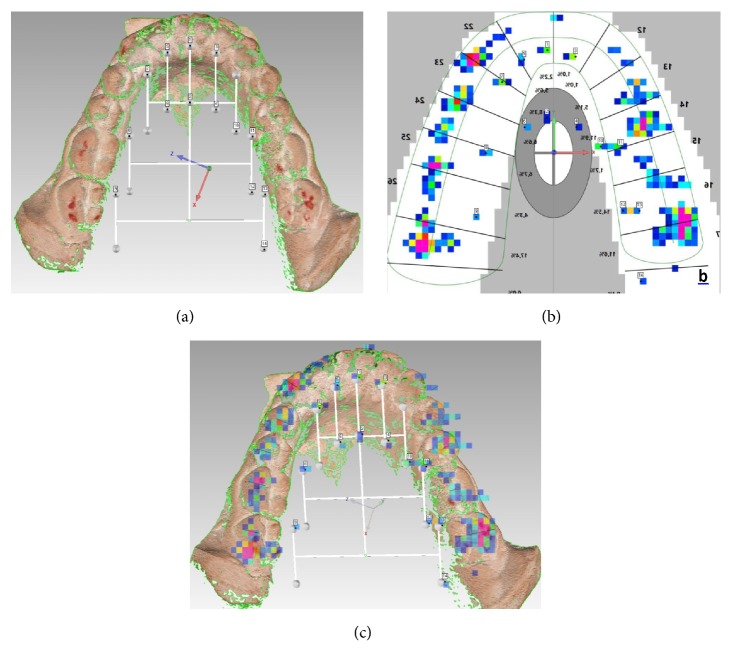
Coincidence quantification. (a) Selection of homologous points in colored digitization. (b) Selection of homologous points in T-Scan's image. (c) Alignment completed; counting of articulating paper marks and matching points.

Finally, after capturing both the patient's contacts and designed reference points with the T-Scan (Figures 3(a) and 3(b)), maximum intercuspation images were exported and aligned with the colored digital casts made in the previous step. The alignment was guided by the reference points, using the same mesh-treatment program (Figures 4(a) and 4(b)). These alignments were visualized with some transparency, allowing precise comparison.

It should be emphasized that the T-Scan's dynamic images must be those of maximum intercuspation, in order to compare both methods with the same quantity of contacts. This is because the articulating paper marks all possible contacts; therefore, the moment of bite that comes closest to that amount of contact area is the maximum intercuspation. In clinical use of this alignment, it should be possible to view the T-Scan timed registration, if necessary.

The accuracy of this method was quantified by searching matches on contacts found by both the T-Scan and articulating paper: the total of detected contacts and the matching colored areas were counted, to obtain some data of interest after a statistical study.

**Figure 5:**
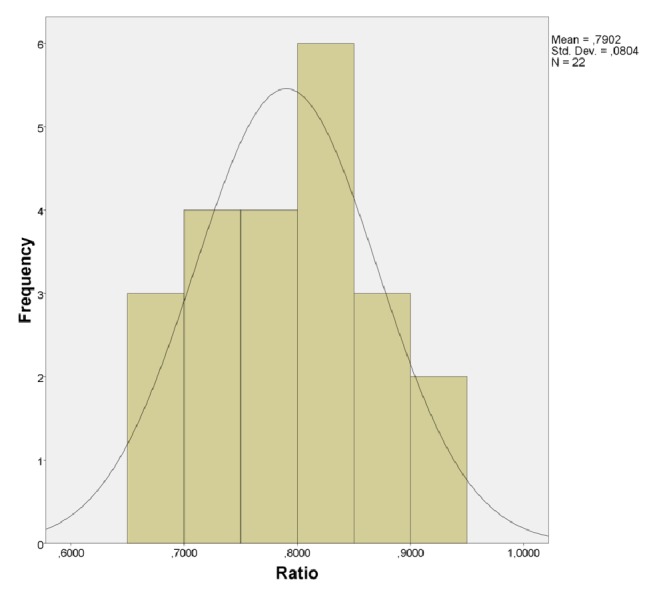
Histogram of distribution.

**Figure 6:**
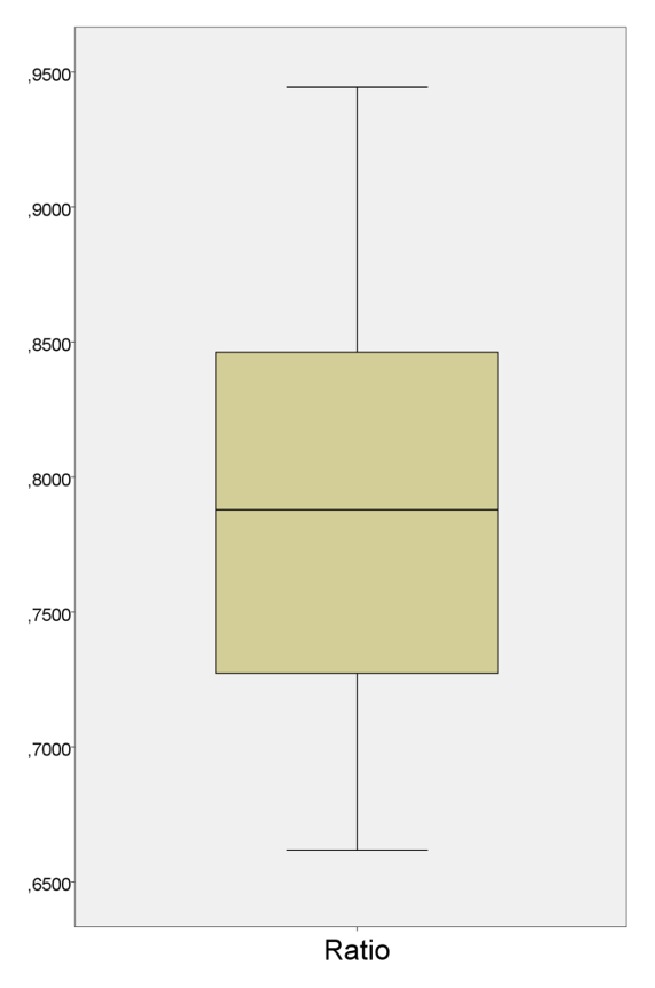
Box-plot.

With such information, the SPSS program was used for statistical calculations.  Table 1 lists the total number of contacts found by the articulating paper method on each scanned part, the contacts detected by the T-Scan which matched the previous ones, and a percentage of contact matching of each case. The distribution of the data is shown in a histogram in Figure 5 and analyzed in Table 1 and Figure 6 with some data of interest, such as the standard deviation, the average, maximum, and minimum, and the grouping of the data around the mean.

## 3. Results and Discussion

As shown in Table 1, the coincidence percentages of T-Scan detected contacts based on articulating paper marks range from 66.18% to 94.44%. They reached a high coincidence percentage on each cast, resulting in a total mean of 79.02%, with a standard deviation of 0.08.

In addition, it can be seen that the normality tests resulted in a significance of 0.610 with the Shapiro–Wilk method (sample size smaller than 50); it therefore has a normal distribution.

The confidence interval demonstrates that, with a reliability score of 95%, the average that other studies of coincidences would achieve would be between 75.46% and 82.59%.

The box-plot in Figure 6 shows that 50% of the closest data to the median are between 72.48% and 84.82%.

Finally, the* Z* score has been calculated. As it indicates, in any alignment following this method there will be an 81.82% probability of a coincidence ratio between 70.83% and 94.44%.

These measurements shed light on the validity of this new alignment method. Even though a greater accuracy of this method could be expected, gathered data reveal a high repeatability in the alignments, which, taking into account this procedure's limitations, shows that it is more reliable than manual alignment because the manual component in the procedure has been greatly minimized and replaced by mathematic methods.

Even so, currently, the practice of combining both T-Scan and articulating paper methods would be recommended to obtain the best results possible. Articulating paper will bring more accuracy to the positioning of occlusal contacts while the T-Scan will show dynamic data and changes in the contacts during the entire bite, which would be impossible to determine if only using an articulating paper.

In addition, measuring accuracy digitally is a step forward and it opens a new range of possibilities. Also, future studies may solve the limitations of this procedure such as possible errors committed in the scanning of dental arches, the prototyping and locating of dental appliances, and implicit limitations of both articulating paper and T-Scan contact detecting systems. In fact, this is the first procedure developed which aligns the T-Scan dynamic data and digital casts methodically; therefore, future investigations or developments will allow an improvement in these figures.

Moreover, even though analyses have been made in previous studies [15–17], such as those on sensitivity or repeatability of the T-Scan, concluding that the correct contact location of the T-Scan exceeds 80%, that patients can be identified, thanks to T-Scan registrations, with probability higher than 90%, and so on, the geometrical location accuracy of contacts has never been measured in the way the present study does, revealing a coincidence ratio of 79.02% mean. Thus, these results are the first that follow the digital dental workflow.

## 4. Conclusions

This study proves the feasibility of the new method developed for the alignment between T-Scan and 3D digital casts (79.02% coincidence ratio mean, with confidence interval between 75.46% and 82.59%), which enables a precise new visualization of the occlusal dynamics.

Although this intuitive visualization is taking its first steps, its advantages are numerous; among others, it will be possible to see the actual force intensity each tooth can withstand, the area covered by each contact accurately, how it changes and moves around the surfaces, and whether some teeth are sharing the charge, and, in general, it gives a better understanding of the occlusal dynamics. Taking this into account, the figures obtained are highly promising because, as aforementioned, future research will be able to enhance these alignments and thus improve its functionality.

## Figures and Tables

**Table 1:** Detected contacts and coincidence rates.

Cases	Arch	Articulating paper contacts	T-Scan coincidences	Coincidence ratio
1	Lower	22	16	72.73%
1	Upper	19	16	84.21%
2	Lower	23	16.5	71,74%
2	Upper	19	14.5	76.32%
3	Lower	13	9.5	73.08%
3	Upper	16	13.5	84.38%
4	Lower	33	25	75.76%
4	Upper	34	22.5	66.18%
5	Lower	30	26.5	88.33%
5	Upper	31	26.5	85.48%
6	Lower	24	20.5	85.42%
6	Upper	26	21	80.77%
7	Lower	13	11	84.62%
7	Upper	12	8.5	70.83%
8	Lower	24	20	83.33%
8	Upper	22	17	77.27%
9	Lower	34	26	76.47%
9	Upper	33	26.5	80.30%
10	Lower	32	21.5	67.19%
10	Upper	26	17.5	67.31%
11	Lower	26	24	92.31%
11	Upper	27	25.5	94.44%

## Data Availability

The data used to support the findings of this study are included within the article.
